# PyLDM - An open source package for lifetime density analysis of time-resolved spectroscopic data

**DOI:** 10.1371/journal.pcbi.1005528

**Published:** 2017-05-22

**Authors:** Gabriel F. Dorlhiac, Clyde Fare, Jasper J. van Thor

**Affiliations:** 1 Department of Life Sciences, Faculty of Natural Science, South Kensington Campus, Imperial College London, London, United Kingdom; 2 Department of Chemistry, Faculty of Natural Science, South Kensington Campus, Imperial College London, London, United Kingdom; Universite de Montreal, CANADA

## Abstract

Ultrafast spectroscopy offers temporal resolution for probing processes in the femto- and picosecond regimes. This has allowed for investigation of energy and charge transfer in numerous photoactive compounds and complexes. However, analysis of the resultant data can be complicated, particularly in more complex biological systems, such as photosystems. Historically, the dual approach of global analysis and target modelling has been used to elucidate kinetic descriptions of the system, and the identity of transient species respectively. With regards to the former, the technique of lifetime density analysis (LDA) offers an appealing alternative. While global analysis approximates the data to the sum of a small number of exponential decays, typically on the order of 2-4, LDA uses a semi-continuous distribution of 100 lifetimes. This allows for the elucidation of lifetime distributions, which may be expected from investigation of complex systems with many chromophores, as opposed to averages. Furthermore, the inherent assumption of linear combinations of decays in global analysis means the technique is unable to describe dynamic motion, a process which is resolvable with LDA. The technique was introduced to the field of photosynthesis over a decade ago by the Holzwarth group. The analysis has been demonstrated to be an important tool to evaluate complex dynamics such as photosynthetic energy transfer, and complements traditional global and target analysis techniques. Although theory has been well described, no open source code has so far been available to perform lifetime density analysis. Therefore, we introduce a python (2.7) based package, PyLDM, to address this need. We furthermore provide a direct comparison of the capabilities of LDA with those of the more familiar global analysis, as well as providing a number of statistical techniques for dealing with the regularization of noisy data.

## Introduction

Ultrafast spectroscopy remains an important tool for the investigation of energy transfer and transiently lived reaction intermediates in photoactive compounds and complexes, due to its high temporal resolution. Analysis of the resultant time-resolved data is often performed with a view to two primary goals. First is the determination of spectral features of transient lived species in order to characterize their molecular state. Second is the extraction of kinetic parameters describing the overall reaction(s). Adoption of new analysis techniques can help provide a better account of the systems under investigation, and complement older ones.

With regards to the first goal, the most prominent technique available is target, or compartment, modelling. In target modelling, a number of compartments, representing molecular states, or a group/average of related molecular states, are assumed. The recorded signal at any given point in time is taken to be the concentration weighted sum of what the spectra of each compartment would be in isolation. A number of kinetic models are tested which determine the time evolution of the concentrations of each compartment. These models may include forward and reversible reactions, as well is independent or parallel decay of various compartments. The fitting procedures then simultaneously optimize the kinetic parameters and the spectra of the indvidual compartments using a variable projection method. For a further review of this technique see [1–5].

While target modelling produces kinetic parameters, historically, the principle method for addressing the second goal, at least at first pass, has been what has been termed global analysis. Global analysis, frequently used with the singular value decomposition (SVD) as a means of dimension reduction, fits all wavelength channels (columns of the data matrix) simultaneously to a sum of a small number of exponential functions [3]. A single set of exponential lifetimes is fit to all wavelengths, while the pre-exponential amplitudes are allowed to vary. This analysis, along with target modelling, gives an overview of the principle decay lifetimes present in the signal. However, a small number of decays may not be sufficient to fully describe the complex kinetics exhibited by non-exponential transients in biological systems of interest such as photosynthetic complexes.

In theory, the data can be fully represented by the integral of a continuous distribution of decays, with the pre-exponential function being the underlying spectral distribution [6–8]. Retrieval of this information would then be as simple as taking the Laplace transform of the signal function; however, as with other sources of time-resolved data, an analytical formulation of the signal is not known in ultrafast spectroscopy. In order to address this, numerical approximations are needed; however, numerical inversion of the Laplace transform is a highly ill-conditioned problem [7]. Lifetime density analysis (LDA), popularized by the Holzwarth group [9] in the field of photosynthesis research, approximates the continuous integral to a semi-continuous sum of *n* = ∼100 lifetimes distributed evenly along the time-scale of the experiment:

$$\Delta A\left(t,\lambda \right)=\sum_{j=1}^{n}x_{j}\left(\tau_{j},\lambda \right)e^{-\frac{t}{\tau_{j}}}⊗IRF\left(t,\lambda \right)$$ (1)

where Δ*A* is the recorded data, and the amplitudes *x*(*τ*, *λ*) represent the probability of a process with the associated lifetime *τ* occuring at the particular wavelength *λ*. This procedure is analogous to the exponential series method developed in the late 1980s and early 1990s [7, 8]. The IRF represents the instrument response function, with ⊗ indicating convolution (see, e.g., [4, 5] for review). Alternatively this can be rewritten in matrix notation as:

$${\vec{A}}_{\lambda }=D{\vec{x}}_{\lambda }$$ (2)

where ${\vec{A}}_{\lambda }$ is the column vector of length *m* containing the ΔOD measurements recorded at *m* time delays, and ${\vec{x}}_{\lambda }$ is the column vector of length *n* containing the pre-exponential amplitudes associated with the *n* lifetimes. *D* is an *m* × *n* matrix of the IRF convolved exponential decays.

The amplitude vectors at all wavelengths λ are compiled into contour maps, or lifetime density maps (LDMs), with lifetime *τ* on the y-axis and wavelength λ on the x-axis. The positive and negative contours correspond to the values of x. The major advantage of this analysis technique is that it provides an immediate overview of the the kinetic distributions present at all wavelengths, and does so in a visually concise way. Furthermore, LDA is able to handle heterogeneous decay lifetimes for single species, and pick up on dynamic motion, two features common to biological data with which global analysis has struggled. Yet despite these advantages, and extensive use of the technique by the Holzwarth group [9–14], widespread adoption has been limited to date (e.g. [15–17]). We believe this to be due to the unavailability of tools performing this type of analysis. The OPTIMUS program [17] recently released a module facilitating this type of analysis; however, the code remains closed-source.

This lack of widespread adoption is particularly striking given the historical motivations leading to the development of the technique. The need for a complement to global analysis began to appear with the advent of more powerful detection schemes. This was particularly true for improved fluorescence detection. Although the earliest time-correlated single photon counting dates back to the 1960s [18], improvements, and greater popularization occurring in the mid 1980s (see e.g. [19]), led to the fitting of more complex models with greater numbers of lifetimes. Additionally, a new concern arose as to whether it was possible to recover kinetic distributions, both in fluorescence spectroscopy, and related fields, modelling time series. As early as the 1960s and 1970s [20] the maximum entropy method was developed as a means to recover these distributions, and was applied to a variety of fields from the 1980s onwards, e.g. [21–24]. A competing approach, the exponential series method, was also developed in the 1980s. The advantages of this method are discussed in comparison to the maximum entropy method by Siemiarczuk et. al. [8]. This technique and observations form the basis of follow-up work by the Holzwarth group [9], upon which the current work is based. The introduction of the use of regularization, a topic discussed at length below, by Landl et. al. [7] was particularly important for the development of this technique in its current form.

We present here an open-source python package, with GUI, to allow for the performance of this type of analysis. We also provide a comprehensive overview of the theory and mathematics behind the approach, and a comparison with the more well-known global analysis.

## Design and implementation

### General remarks

All code was written in python 2.7. The goal was to provide a package that allowed the user to experiment with a number of different options and methods in order to produce the best LDMs given their dataset. At the same time, visual inspection throughout the fitting process was emphasized, while also providing a number of statistical measures to assist in avoiding over-fitting and model bias. Finally, the package was designed to use as few dependencies as possible, to allow for easier installation and code inspection. As such, only the relatively commonplace NumPy [25], SciPy [26], and Matplotlib [27] packages were used. A full manual for installation and use is available with the software.

### Global analysis and singular value decomposition

In order to best demonstrate the utility of LDA, a comparison with the older, and more developed technique of global analysis is provided. To determine the number of exponential decays to include in the analysis, loaded datasets can have their SVD visually inspected. The SVD of the *m* × *n* data matrix *A* containing spectroscopic measurements recorded at *m* time delays, across *n* wavelength channels is given by:

$$A=USV^{T}$$ (3)

where *U* is the *m* × *m* unitary matrix of left singular vectors giving the temporal dependence of the signal, *V*^*T*^ is the *n* × *n* unitary matrix giving the spectral dependence of the signal, and *S* is the *m* × *n* diagonal matrix of singular values.

Typically the number of singular values significantly larger than the rest is taken to be the number of independently resolvable lifetimes in a global analysis [3]. This number of exponential decays is fit simultaneously to an equal number of weighted left singular vectors, that is, the columns of *U* weighted by their corresponding singular values. This process serves to reduce the dimensionality of the problem. Note, however, that the number of singular values chosen reflects only an estimate of the number of orthogonal components appropriate to represent the data, and makes no estimation of any underlying reaction scheme. Additionally, large noise components can have singular values larger than small signal components [5]. In order to address this, the weighted left singular vectors can be visually inspected before fitting. Those corresponding to signal components should have a regular structure. In the case that a left singular vector corresponding to a smaller singular value has more regular structure than that corresponding to a larger one, it can be used in the fitting procedure in its stead, at the users discretion. For example, if there are four large singular values, but the 5th left singular vector has more regular structure than the 4th, the first, second, third and fifth vectors can be used for fitting.

We begin the fitting with an equation similar to eq 2, substituting however, the weighted left singular vectors for the full data matrix:

$${\left(US\right)}_{n}=D\left(\vec{\tau }\right)x$$ (4)

where the (*US*)_*n*_ represents the matrix of chosen weighted left singular vectors. Note, however, that in this case *D* will be an *n* × *n* square matrix, where *n* is the number of lifetimes, and that it is itself a function of a parameter vector of lifetimes, $\vec{\tau }$. In addition, *x* is also a matrix corresponding to the pre-exponential function for each weighted left singular vector.

Determination of *x* and the associated lifetimes is done by solving the associated non-linear least squares problem:

$$\min_{x,\vec{\tau }}{∥D\left(\vec{\tau }\right)x-{\left(US\right)}_{n}∥}_{2}^{2}$$ (5)

A variable projection method [28] is employed in conjunction with SciPy’s implementation of the Levenberg-Marquardt algorithm. Solely the vector of lifetimes, $\vec{\tau }$, is fed to the solver. At each iteration, the solution for *x* is found by taking the QR-decomposition of *D* and solving using back substitution.

The results of the fit can be inspected, and redone with new initial guesses and bounds if found to be divergent, and the lifetimes are stored for mapping onto the LDMs.

### Lifetime density analysis

Unlike in global analysis, the pre-exponential function itself is what is of interest in LDA. However, solving for $\vec{x}$ in eq 2, or the corresponding matrix of pre-exponential functions at all wavelengths, requires solution through the associated least-squares minimization, as before. In this case though, as the number of lifetimes, i.e. parameters, is vary large, over-fitting of the data is a bigger concern. The initial discretization producing eq 1, helps to improve the conditioning of the inverse problem; however, as the number of lifetimes is increased to find a meaningful approximation to the underlying spectral distribution, this conditioning deteriorates [7]. In order to address this, regularization is introduced. Regularization is the process whereby the objective function is modified to penalize large coefficients of fitting, in this case $\vec{x}$. While the unregularized least squares problem will find the mathematically optimal solution, this is undesirable, as the large number of fitting parameters will result in fits of the noise, making the solution extremely sensitive to perturbations. Regularization introduces bias to the fit in an attempt to avoid this. As a result it is effectively smoothing the data. A number of regularization procedures and penalty terms exist, all imposing different assumptions on the data. Those included in the software package will be discussed in turn below. For a thorough interpretation of these techniques in a Bayesian framework, see [29].

#### Truncated SVD

The truncated SVD was proposed by Per Hansen in 1987 [30] as an alternative to the more well-known Tikhonov regularization (discussed below). A rank *K* approximation is used for the design matrix, in this case the matrix of exponential decays, where all the small singular values beyond some cut-off *K* are replaced with 0. This means that the higher frequency components of the pre-exponential amplitudes are ignored [30]. The major difference with this technique compared to Tikhonov regularization is that while the latter also dampens the components corresponding to the smallest singular values, it does so with a smooth filter function compared to the step-function used here. We note that while this technique may produce solutions similar to those of Tikhonov regularization, its use for inverse problems where the matrix has ill-determined numerical rank (such as in this case of inverse Laplace transformation) may produce solutions sensitive to perturbation [30]. It is therefore suggested only as a technique to provide a rough overview of kinetic distributions, early on in analysis.

Eq 2 is rewritten with the truncated SVD for *D*:

$${\vec{A}}_{\lambda }=US_{K}V^{T}{\vec{x}}_{\lambda }$$ (6)

which when solved for ${\vec{x}}_{\lambda }$ yields:

$${\hat{x}}_{\lambda }^{K}=VS_{K}^{-1}U^{T}{\vec{A}}_{\lambda }$$ (7)

as the inverses of the unitary matrices *U* and *V*^*T*^ are simply their transposes.

Solving for the LDMs in this manner requires only the use of the SciPy singular value decomposition routine. Note that this regularization technique should not be confused with low rank data approximation, which applies a similar cut-off filter to the *data matrix* *A* as a means of dimension reduction or compression of large matrices. This feature is also included in the GUI.

#### Matrix regularization: Tikhonov - *ℓ*_2_

The most well known regularization method, and the one that has likely been in use the longest, is Tikhonov regularization, alternatively known as *ℓ*_2_ regularization, or ridge regression [31–33]. Tikhonov regularization imposes a direct penalty on the amplitudes of the regression coefficients in this case the values of *x* in eq 2. This type of regularization is one of a broader category matrix regularizations, with the penalty in this case taking the form of a 2-norm. The associated Tikhonov regularized least squares minimization problem then becomes:

$$\min_{\vec{x}}∥D{\vec{x}}_{\lambda }-{\vec{A}}_{\lambda }{∥}_{2}^{2}+\alpha {∥L{\vec{x}}_{\lambda }∥}_{2}^{2}$$ (8)

where L is a matrix which in standard form is the identity. Depending on the structure of the data set a number of alternative matrices may be used, including 1st and 2nd derivative approximations, as well as a “fused” [36] identity and 1st derivative matrix. All of these options are available to the user; however, the use of the identity matrix imposes the fewest assumptions on the fitting procedure and is used by default. For a review on some of the situations where the other matrices are appropriate see [33, 34]. *α* is a positive valued hyper-parameter which represents the trade-off between minimization of the residuals and the regression coefficients. For noisier data one would expect the value providing appropriate results to increase. A number of ways of selecting the *α* value are discussed below.

Through the use of augmented matrices, the Tikhonov regularized minimization problem can be reduced to an ordinary least squares problem. We define $\tilde{D}=(\begin{array}{c} D\\ \sqrt{\alpha }L \end{array})$ and $\tilde{A}=(\begin{array}{c} A\\ 0 \end{array})$. Eq 8 then becomes:

$$\min_{\vec{x}}{∥\tilde{D}{\vec{x}}_{\lambda }-{\tilde{A}}_{\lambda }∥}_{2}^{2}$$ (9)

Eq 9 has the closed form solution, for a given *α* value:

$${\hat{x}}_{\lambda }^{\alpha }={\left({\tilde{D}}^{T}\tilde{D}\right)}^{-1}\tilde{D}{\tilde{A}}_{\lambda }$$ (10)

To avoid instability associated with numerical inversion, the SVD is again used. The tildes only indicate that the SVD was taken for $\tilde{D}$ and not *D*. The final result is:

$${\hat{x}}_{\lambda }^{\alpha }=\tilde{V}{\tilde{S}}^{-1}{\tilde{U}}^{T}{\tilde{A}}_{\lambda }$$ (11)

The solution is found for a user specified range of alpha values, allowing the user to cycle through all the resultant maps. In addition the various *α* selection statistics are also displayed. The user can choose to display these assuming a single *α* for all wavelength channels, or allowing separate statistics to be calculated for each wavelength channel independently. The latter choice may be desired if there is some regular structure to the noise in the data across wavelength channels; however, a single *α* is likely sufficient.

Tikhonov regularization is useful when there is little known about the underlying kinetic distributions. The imposed penalty term assumes that $\vec{x}$ and the noise in the data are normally distributed [29]. This will produce results with somewhat broader kinetic distributions about key lifetime contributions. We note that it is therefore difficult to separate two near-lying kinetic distributions, or a single lifetime from a nearby distribution.

#### Matrix regularization: LASSO - *ℓ*_1_ and elastic net

A second form of matrix regularization, called the LASSO, was introduced by Robert Tibshirani in 1994 [35], substituting a 1-norm for the 2-norm. The LASSO was originally studied for the case of *L* = *I*; however, Robert Tibshirani later introduced what is known as the “fused” [36], and generalized [37] LASSOs as alternatives. One possible advantage of these methods over Tikhonov regularization is that while the former will shrink regression coefficients, it will not set any to zero, while the LASSO will [35]. This allows for further variable selection, making the differences between the pre-exponential amplitudes for various associated lifetimes greater. This is because the 1-norm penalty term assumes that $\vec{x}$ is drawn from a Laplacian distribution [29], producing sparser solutions. This assumption is a good one if the data is expected to be described by few, narrowly distributed lifetimes. However, the minimization problem:

$$\min_{\vec{x}}∥D{\vec{x}}_{\lambda }-{\vec{A}}_{\lambda }{∥}_{2}^{2}+\alpha {∥L{\vec{x}}_{\lambda }∥}_{1}$$ (12)

does not have a closed form solution unless the columns of the matrix *D* are orthogonal, in which case the solution for each entry *j* in ${\vec{x}}_{\lambda }$ is (SI for derivation):

$${\hat{x}}_{j}^{\alpha }=sgn\left({\hat{x}}_{j}^{OLS}\right)\left(2\right|{\hat{x}}_{j}^{OLS}{\left|-\alpha \right)}^{+}$$ (13)

where the superscript *OLS* indicates the ordinary least squares solution, and (⋅)^+^ indicates the maximum between the value within the parentheses and 0.

Unfortunately the case of an orthogonal regression matrix is very rare, and so a number of different algorithms have been proposed for solving the LASSO regularization problem. In a paper in 2016 [38], Shifeng Xiong et al. introduced a new algorithm based on the principle of orthogonalizing the regression matrix through the addition of new rows. The responses are then imputed at each iteration of the algorithm. However, neither the rows, nor the responses need to be explicitly computed (see [38] for derivation). This algorithm was chosen for this package and is reproduced below:

Algorithm

1. Set the initial guess for ${\hat{x}}_{\lambda }$ equal to the Tikhonov regularized solution

2. Compute *g* = *γ*_1_ where *γ*_1_ is the largest eigenvalue of *D*^*T*^ *D*

3. Set *B* = *gI*_*p*_ − *D*^*T*^ *D* where *I*_*p*_ is the *p* × *p* identity matrix, where *p* is the number of parameters

4. Set *K* = 0 where *K* is the iteration counter

5. for each coefficient *j* in $\hat{x}$

 *while* $\frac{x_{j}^{\left(K\right)}-x_{j}^{\left(K-1\right)}}{x_{j}^{\left(K-1\right)}}>10e-12\wedge x_{j}^{\left(K-1\right)}\neq 0$

 (a) *K* = *K* + 1

 (b) $U^{\left(K\right)}=D^{T}{\vec{A}}_{j}+B_{j}x^{\left(K-1\right)}$

 (c) $x^{\left(K\right)}=sgn\left(U\right){\left(\frac{\left|U\right|}{g}-\alpha \right)}^{+}$

A combination of the LASSO and Tikhonov regularization, called the elastic net, was introduced in 2005 by Hui Zou [39], and is also made available in this package. It was shown to outperform the LASSO technique while maintaining similar variable selection properties, particularly in the case where the number of parameters was greater than the number of observations, a situation likely to be encountered by users of this package [39]. The elastic net makes use of both the 1-norm and 2-norm, with the associated minimization problem being:

$$\min_{{\vec{x}}_{\lambda }}∥D{\vec{x}}_{\lambda }-{\vec{A}}_{\lambda }{∥}_{2}^{2}+\alpha (\rho ∥L{\vec{x}}_{\lambda }{∥}_{1}+\left(1-\rho \right)∥L{\vec{x}}_{\lambda }{∥}_{2}^{2})$$ (14)

The additional hyper-parameter *ρ* is introduced to weight the use of the two different norms. When *ρ* = 1 the problem reduces to an ordinary LASSO problem, and likewise when *ρ* = 0, it reduces to a Tikhonov regularization problem. In the intermediate scenario, the elastic net can be reduced to an augmented LASSO problem similarly to how the Tikhonov problem can be reduced to an ordinary least squares one. We define *α*_1_ = *αρ*, *α*_2_ = *α*(1 − *ρ*) and $\tilde{\alpha }=\frac{\alpha_{1}}{\sqrt{1+\alpha_{2}}}$. Our augmented matrices then become:

$$\tilde{D}=\frac{1}{\sqrt{1+\sqrt{\alpha_{2}}}}\left(\begin{array}{c} D\\ \sqrt{\alpha_{2}}L \end{array}\right)$$

$$\tilde{A}=\left(\begin{array}{c} A\\ 0 \end{array}\right)$$

and so our LASSO problem, solved using the algorithm above, is:

$$\min_{{\vec{x}}_{\lambda }}=∥\tilde{D}{\vec{x}}_{\lambda }-{\tilde{A}}_{\lambda }{∥}_{2}^{2}+\tilde{\alpha }{∥L{\vec{x}}_{\lambda }∥}_{1}$$

The solution to the above problem, ${\hat{x}}_{naive}$ is reweighted by (1 + *α*_2_) to give the final elastic net solution, for a given *α* and *ρ* value [39].

The elastic net provides solutions intermediate between the LASSO and Tikhonov regularization. Its use is appropriate when broad distributions are not expected, but LASSO solutions prove unexpectedly sparse.

#### Matrix regularization: Selecting hyper-parameters

While frequently ad-hoc methods, based on visual inspection, are used to select hyper-parameter values, the PyLDM package includes a number of features to assist the user in making the appropriate choice. As a first step, the lifetimes from the global analysis help with visual inspection. Tikhonov regularization has had the longest history, and so the greatest number of hyper-parameter selection techniques have been developed for it. When Tikhonov regularization is used three additional statistics are available to guide the user:

1. The Generalized Cross-Validation (GCV) Statistic [40]
  The optimum *α* value is given by the minimizer of:
  $$GCV\left(\alpha \right)=\frac{∥D{\hat{x}}_{\alpha }{-A∥}_{2}^{2}}{tr{\left(I-H\right)}^{2}}$$
  where *H* = *D*(*D*^*T*^ *D* + *αL*^*T*^ *L*)^−1^ *D*^*T*^, and *tr*(⋅) indicates the trace of the matrix.
2. The *C*_*p*_ statistic [41]
  The optimum *α* value is given by the minimizer of:
  $$C_{p}\left(\alpha \right)={∥D{\hat{x}}_{\alpha }-A∥}_{2}^{2}+2\sigma^{2}\left(df\right)$$
  where *σ*^2^ is the error variance, an estimate of which is given by the error variance of the most complicated model, i.e. the one with the lowest *α* value, and *df* is the degrees of freedom given by the trace of *H* from the previous equation.
3. The L-curve [42, 43] The L-curve is a plot of the smoothing norm $∥L\hat{x}{∥}_{1,2}=S$, in either the Tikhonov or LASSO cases, vs the residuals, $∥D{\hat{x}}_{\alpha }{-A∥}_{2}^{2}=R$. Each solution at a given *α* value corresponds to a single point on the graph, and the L-curve estimate for the ideal solution is given by the *α* value corresponding to the point of maximum curvature, given by the formula:
  $$\max_{\alpha }\kappa \left(\alpha \right)=\frac{\frac{dR}{d\alpha }\frac{d^{2}S}{d\alpha^{2}}-\frac{d^{2}R}{d\alpha^{2}}\frac{dS}{d\alpha }}{{\left({\left(\frac{dR}{d\alpha }\right)}^{2}+{\left(\frac{dS}{d\alpha }\right)}^{2}\right)}^{3/2}}$$

Fewer statistics are currently available for selection of hyper-parameters when using the LASSO, particularly due to the lack of differentiability; however, the Cp statistic has been suggested for LASSO-based regularization [41], as has a modified L1-curve [43].

## Results

**Fig 1:**
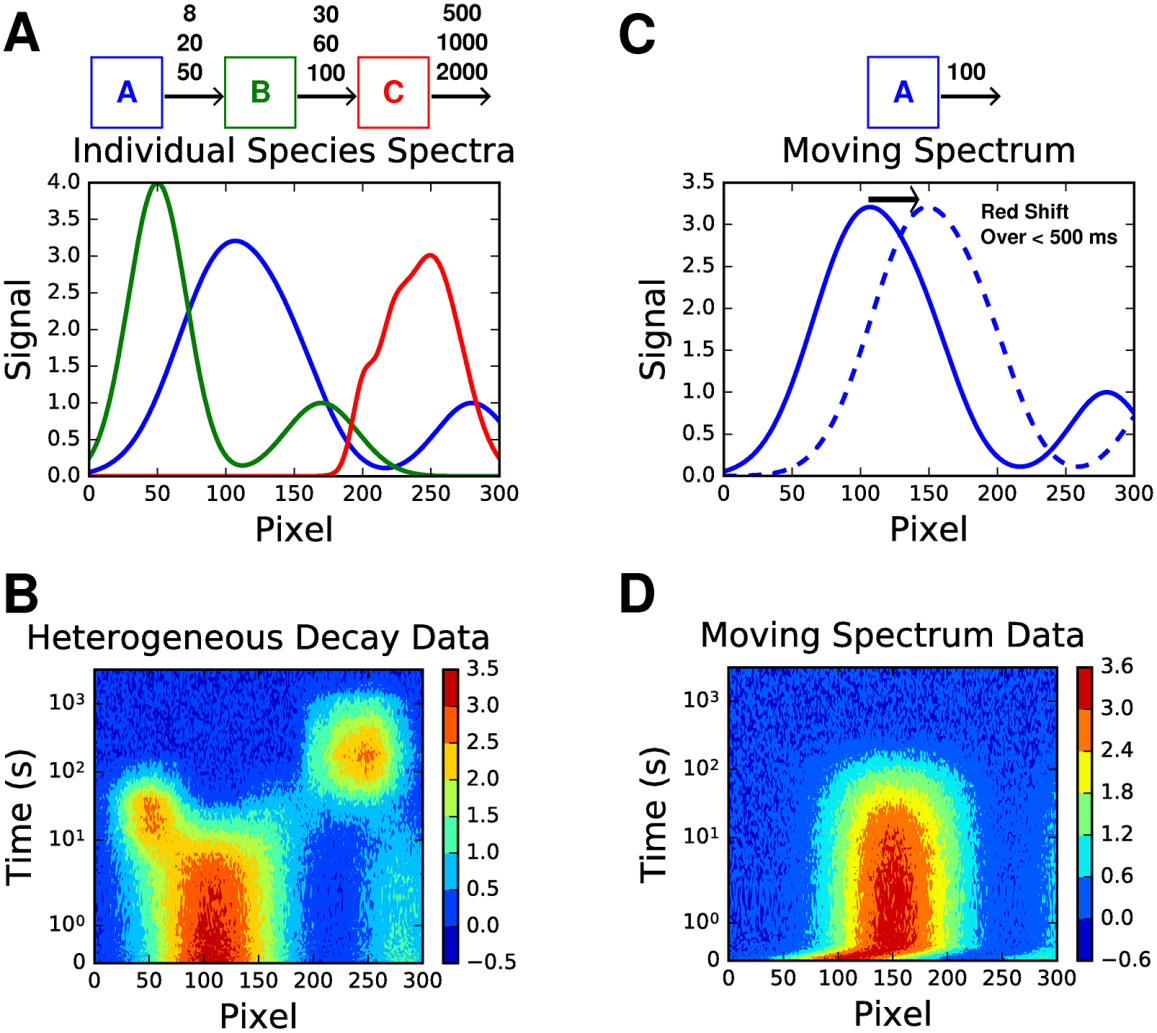
Heterogeneous decay and dynamic motion data sets. **A,C** Species spectra and kinetic schemes used to construct the heterogeneous decay and dynamic motion data sets respectively. All decay lifetimes are indicated in seconds. For A, each species had an equal chance of decaying with any of the three lifetimes at each time point. **B,D** The respective data sets for heterogeneous decay, and dynamic motion, displayed as contour maps. Both have had 10% random noise added. Note that the y-axis scale is a lin-log scale, i.e. linear between 0 and 1, and log from there on.

The above methods have been recently applied to analyse measurements of photosynthetic energy transfer and charge separation in photosystem I(submitted). For the purposes of illustration, three synthetic data sets with 10% random noise were created. To begin, two simpler data sets (Fig 1B and 1D) are presented to more easily illustrate the ability of LDA to resolve heterogeneous decay, and dynamic motion, respectively, before moving on to a more complex scenario.

**Fig 2:**
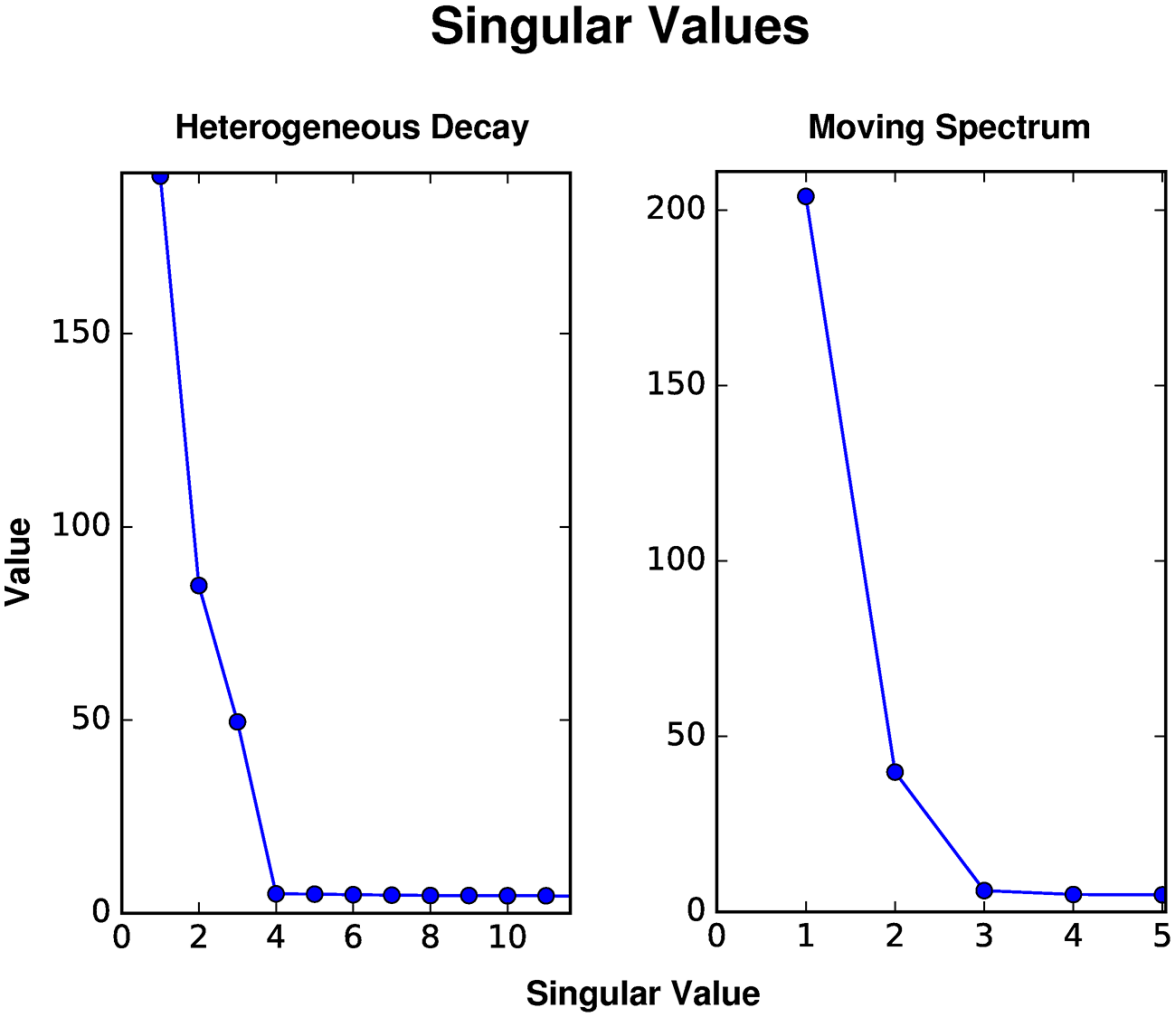
Singular values. The singular values for the heterogeneous decay and dynamic motion data sets in Fig 1B and 1D. Note that the heterogeneous decay data has three significant values and the dynamic motion data has two.

Beginning with the heterogeneous decay scenario, whose kinetic scheme is given by Fig 1A, we observe that an analysis of the SVD indicates only three principle components (Fig 2). This suggests that a global analysis will result in at most three lifetimes, in comparison to the nine that determine the reaction scheme (S1 Fig for fits). The SVD of the dynamic motion scenario, given by the red-shift of a single species shown in Fig 1C, shows that there are potentially two lifetimes that could be resolved by a global analysis (Fig 2). This demonstrates that the SVD acts to approximate the time dependent measurements with linear combinations of basis spectra that in fact do not represent the spectral dynamics. Instead, LDA may be used to analyze such dynamic behavior. Furthermore, we note that the major component, corresponding to the first singular value, is well fit by a single decay (S2 Fig).

**Fig 3:**
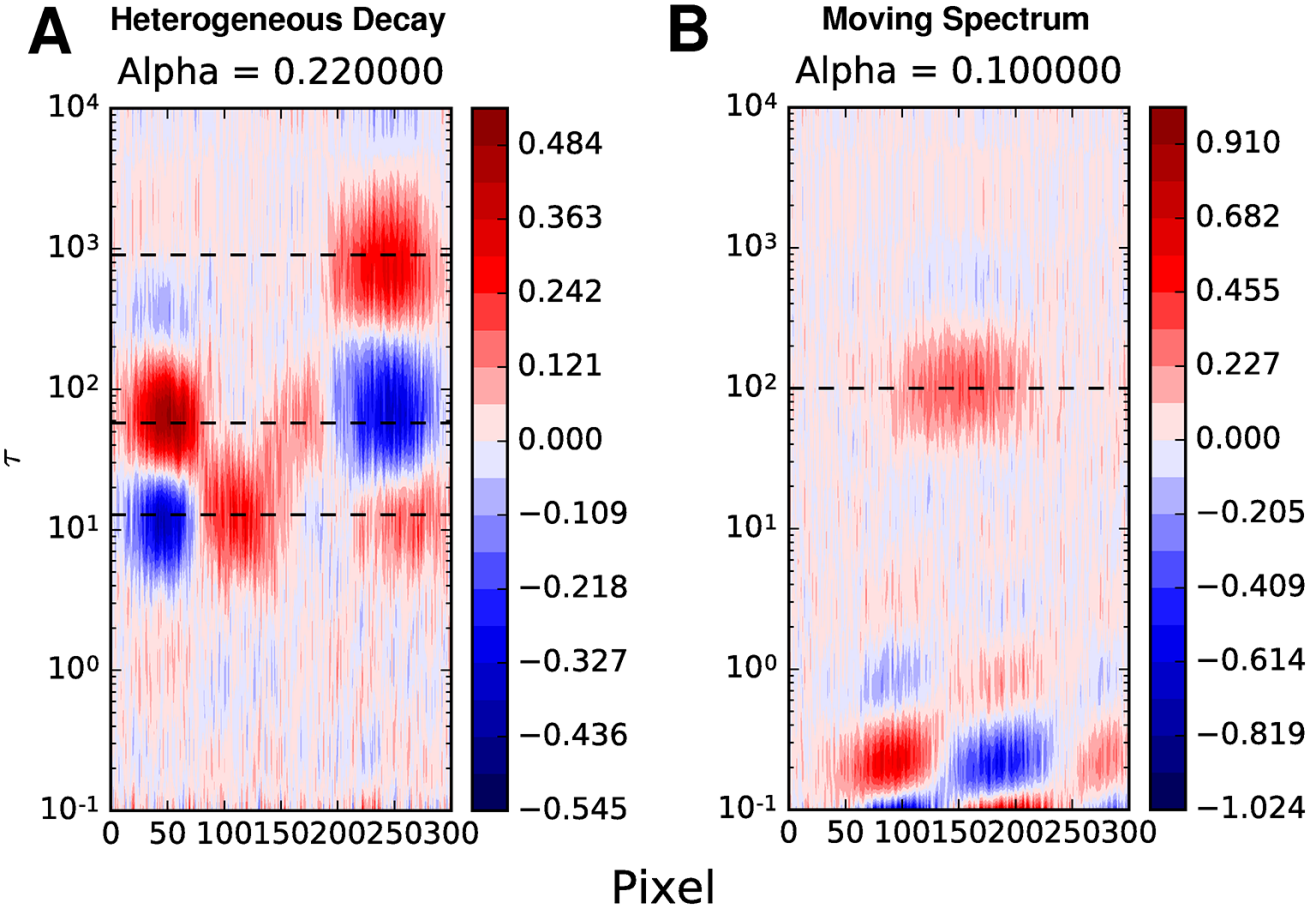
LDMs for heterogeneous decay and dynamic motion data. The Tikhonov regularized lifetime maps for the heterogeneous decay (**A**) and dynamic motion (**B**) data. The dashed horizontal lines indicate the lifetimes determined from global analysis. The indicated alpha values were selected using a compromise between suggested values by the GCV and Cp statistic.

The use of Tikhonov, LASSO and Elastic Net regularization routines produced similar results for these data sets, and for brevity only the Tikhonov results will be shown (Fig 3). The lifetimes resulting from the global analysis of the heterogeneous decay data (12 s, 58 s, 905 s) while within the range of lifetimes used, did not coincide with any of them. The LDM on the other hand displayed broader peaks that were representative of the entire range of decay lifetimes. Note, however, that some vertical broadening of peaks can be expected, particularly with Tikhonov regularization. This is most apparent by observing that multiple smaller amplitudes will reduce ${∥x∥}_{2}^{2}$ compared to a single large amplitude. From a probabilistic perspective, this is a consequence of the assumption of normally distributed amplitudes [29]. This is evident from the LDM of the dynamic motion data, where the central peak at 100 s is significantly broader than would be expected. Furthermore, while one might anticipate horizontal broadening of the 100 s lifetime due to decay while the species’ maximum absorption was at various wavelengths, it is not a large amount, as there are many more time points while the species is at its red-shifted position, compared to the initial one.

**Fig 4:**
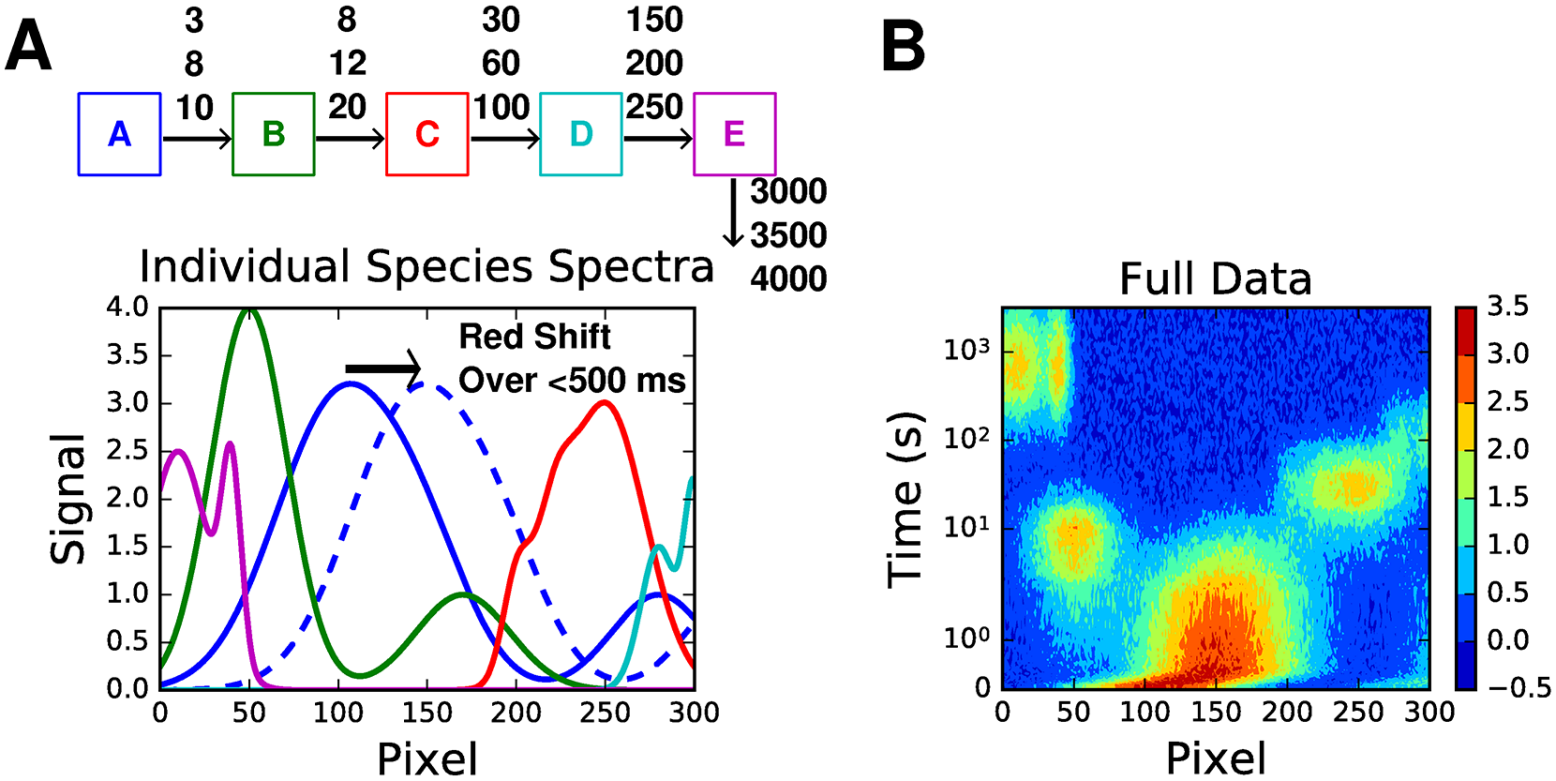
Combined data set. A combined data set comprising dynamic motion and heterogeneous decay, with five species. **A** The kinetic scheme and individual species spectra used to construct the data. The lifetimes are in seconds, and each species had an equal chance of decaying with any of the lifetimes indicated. **B** A contour map showing the resultant data. 10% random noise has been added to the data. Note that the y-axis scale is a lin-log scale, i.e. linear between 0 and 1, and log from there on.

**Fig 5:**
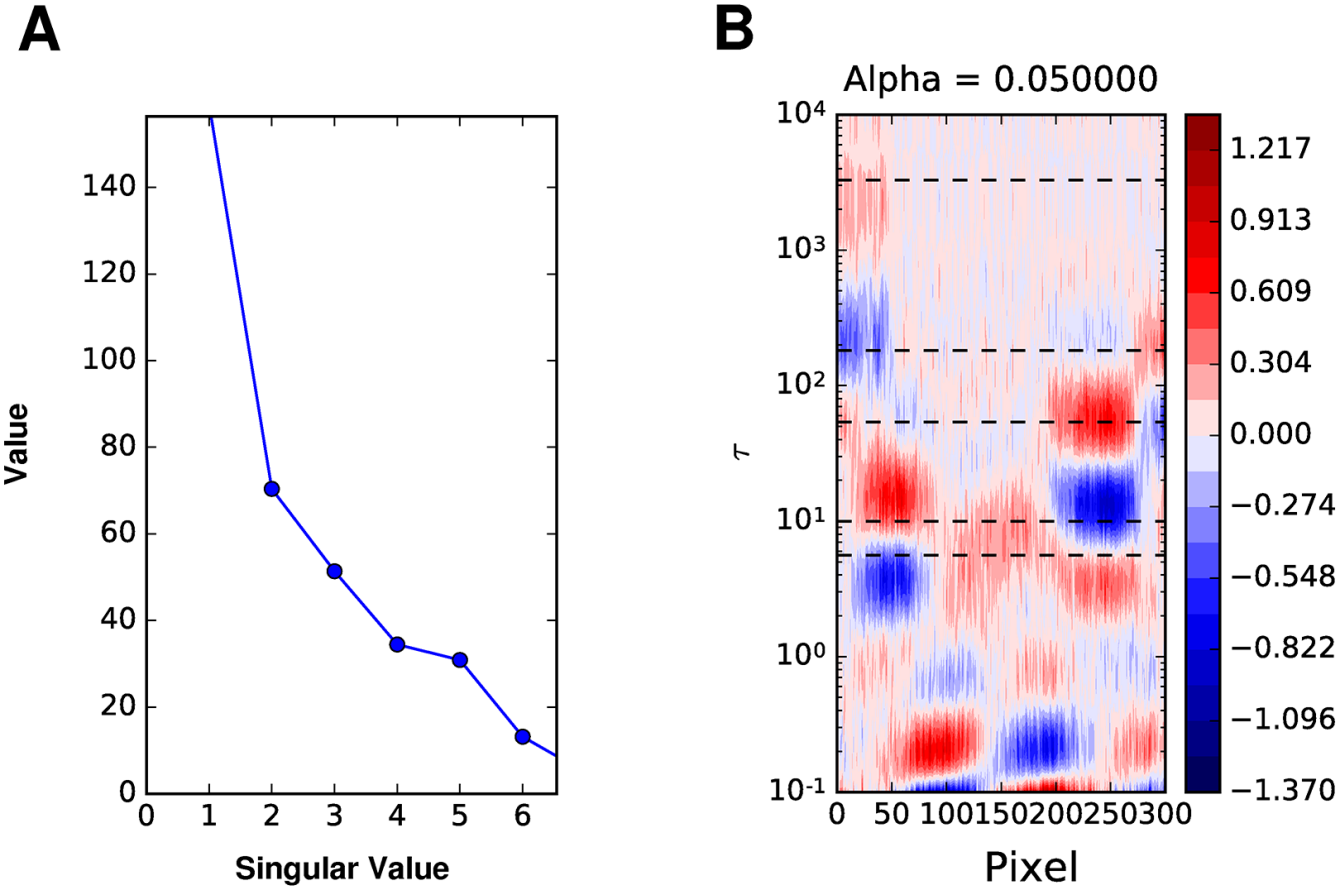
Singular values and LDM for combined data. **A** The singular values from the SVD of the data shown in Fig 4B. Note that there are 6 significant values. **B** The Tikhonov regularized LDM. The dashed horizontal lines indicate lifetimes determined from global analysis.

Combining these types of features, we present a five species reaction, with heterogeneous decay lifetimes, and dynamic motion (Fig 4). Such a scheme produces data sufficiently complex to approximate the types of electronic spectra that may be expected from experiments involving many chromophores, such as in photosystem I/II. Note that an analysis of the singular values (Fig 5A) also indicates the potential for 6 lifetimes. Additionally, problems were encountered regarding convergence for the fitting routines when fitting 6 components.

As before, the LASSO and elastic net solutions were similar to the Tikhonov solutions for low *α* values, and so only the latter will be presented (Fig 5B). However, to illustrate their behaviour a comparison is provided in S3 Fig.

Despite the complexity of the data, the LDM retains clearly defined peaks, which correspond well to the lifetime distributions from the kinetic scheme, particularly at shorter lifetimes. The slight divergence at longer lifetimes is expected given that these lifetimes are longer than the longest time points in the data set.

## Availability and future directions

The software is freely available for download from www.github.com/gadorlhiac/pyldm, along with the instruction manual and datasets. Also included is an IPython notebook used for the creation of the data sets. Future versions of the software will focus on expansion of available statistics for the LASSO, truncated SVD and elastic net. Possibilities for this include implementation of a discrete picard condition [44] for the truncated SVD, and a two dimensional selection model as described in [45]. Additionally, integration with routines for target modelling may be pursued.

## Supporting information

S1 Fig Global analysis fits of heterogenous decay data. **A** Decay associated difference spectra for the three lifetimes fit to 3 wLSVs. **B** The three wLSVs and the corresponding fits.
(TIF)

S2 Fig Global analysis fits of dynamically moving band. **A** Fit of a single wLSV using one lifetime. The fitted lifetime corresponded to 99.9 s. **B** The decay associated difference spectrum and fit of the second wLSV when using two wLSVs and two lifetimes. Note the divergence in the fit. The initial guesses were 5 and 100 s. Only initial guesses very close to the actual values of the lifetimes converged (e.g. guesses of .2 and 100 s).
(TIF)

S3 Fig Comparison of LDMs. **A-D** LDMs produced with the indicated regularization routines. Alpha values and K for truncated SVD are also noted.
(TIF)

S1 Appendix (PDF)
