# Supplementary figures and images for: PyLDM - An open source package for lifetime density analysis of time-resolved spectroscopic data
Source: PLoS Comput Biol. 2017 May 22;13(5):e1005528. doi: 10.1371/journal.pcbi.1005528 (PMC5460884; doi:10.1371/journal.pcbi.1005528)

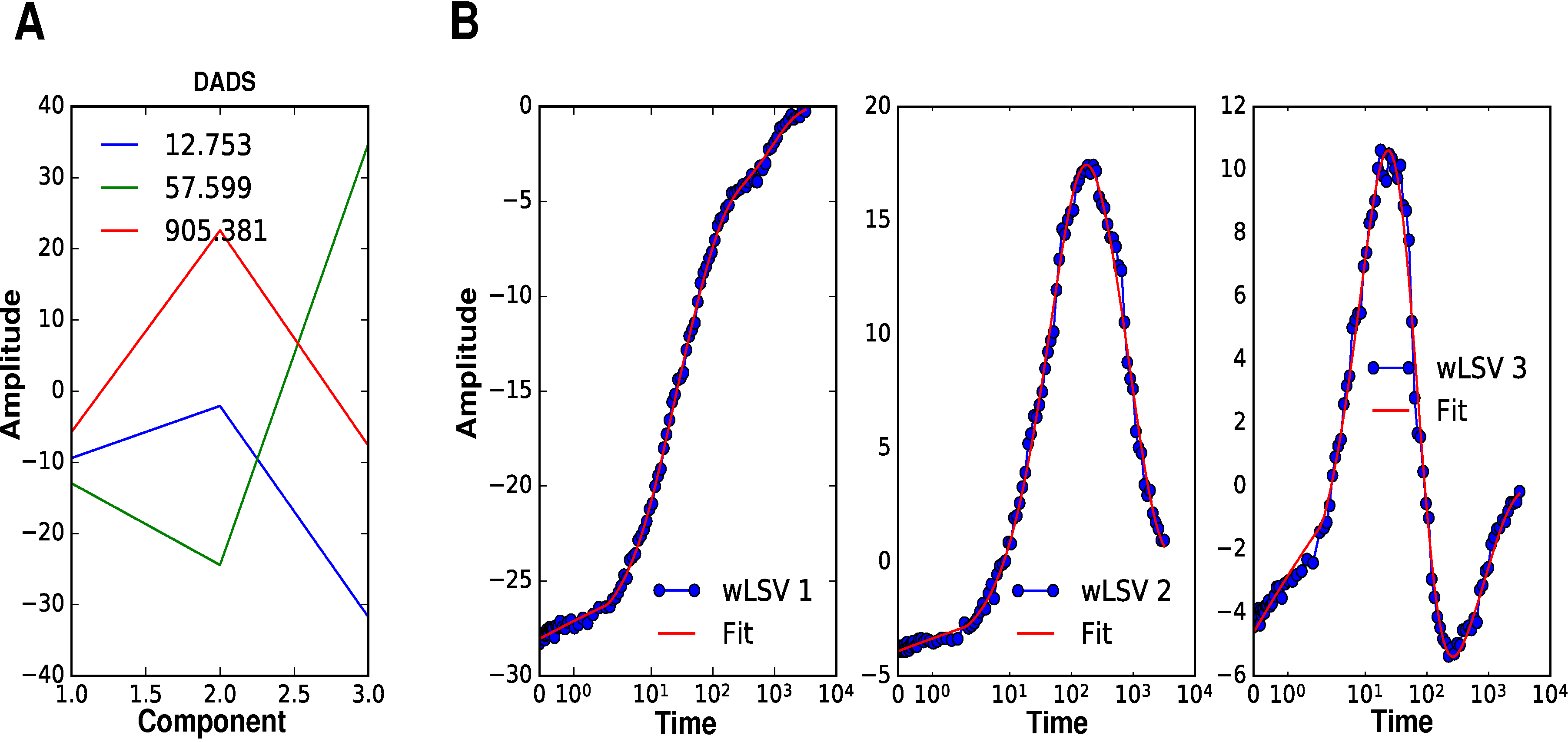

Supplement: S1 Fig — A Decay associated difference spectra for the three lifetimes fit to 3 wLSVs. B The three wLSVs and the corresponding fits. (TIF) [file pcbi.1005528.s001.tif]

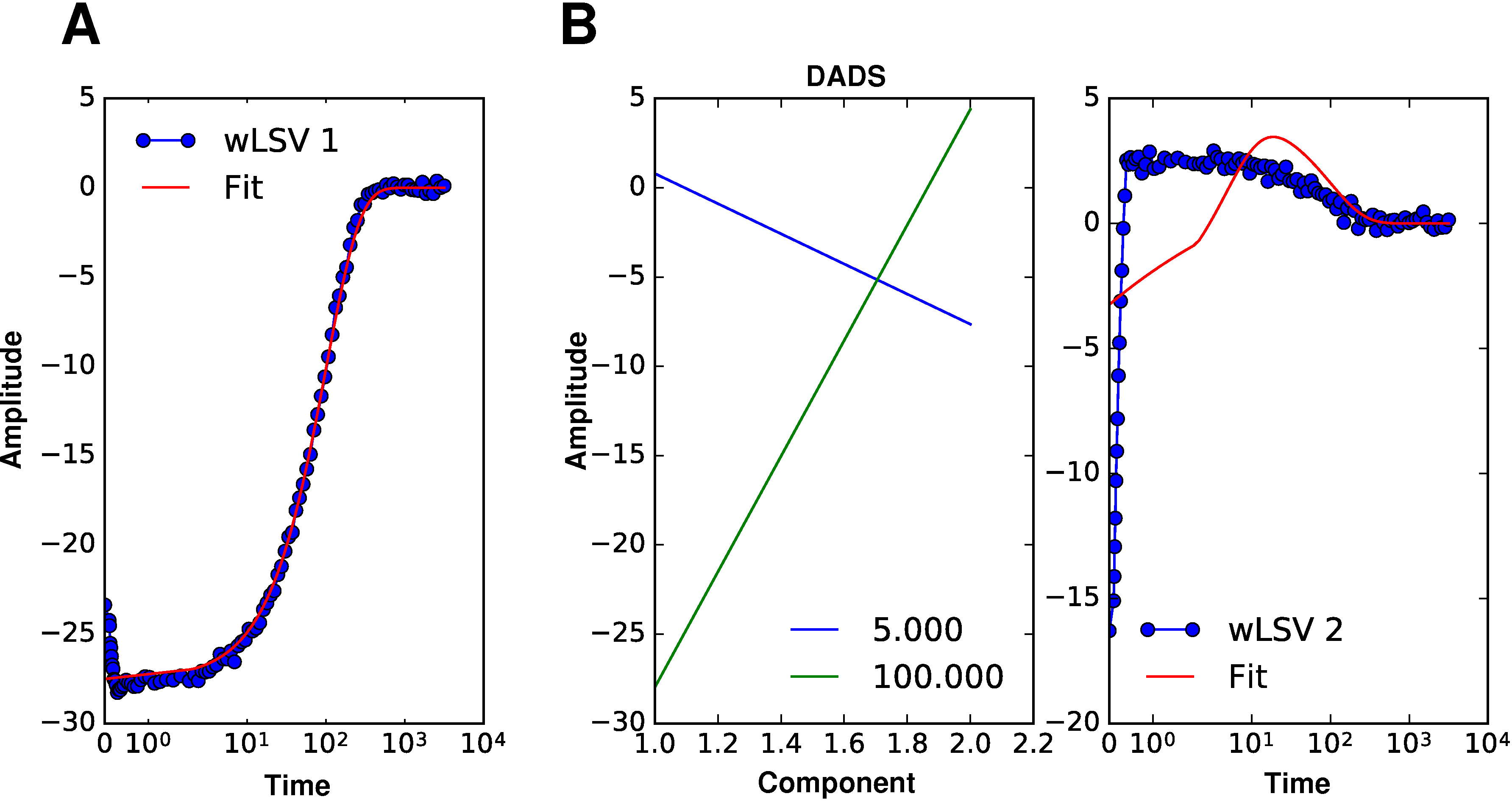

Supplement: S2 Fig — A Fit of a single wLSV using one lifetime. The fitted lifetime corresponded to 99.9 s. B The decay associated difference spectrum and fit of the second wLSV when using two wLSVs and two lifetimes. Note the divergence in the fit. The initial guesses were 5 and 100 s. Only initial guesses very close to the actual values of the lifetimes converged (e.g. guesses of .2 and 100 s). (TIF) [file pcbi.1005528.s002.tif]

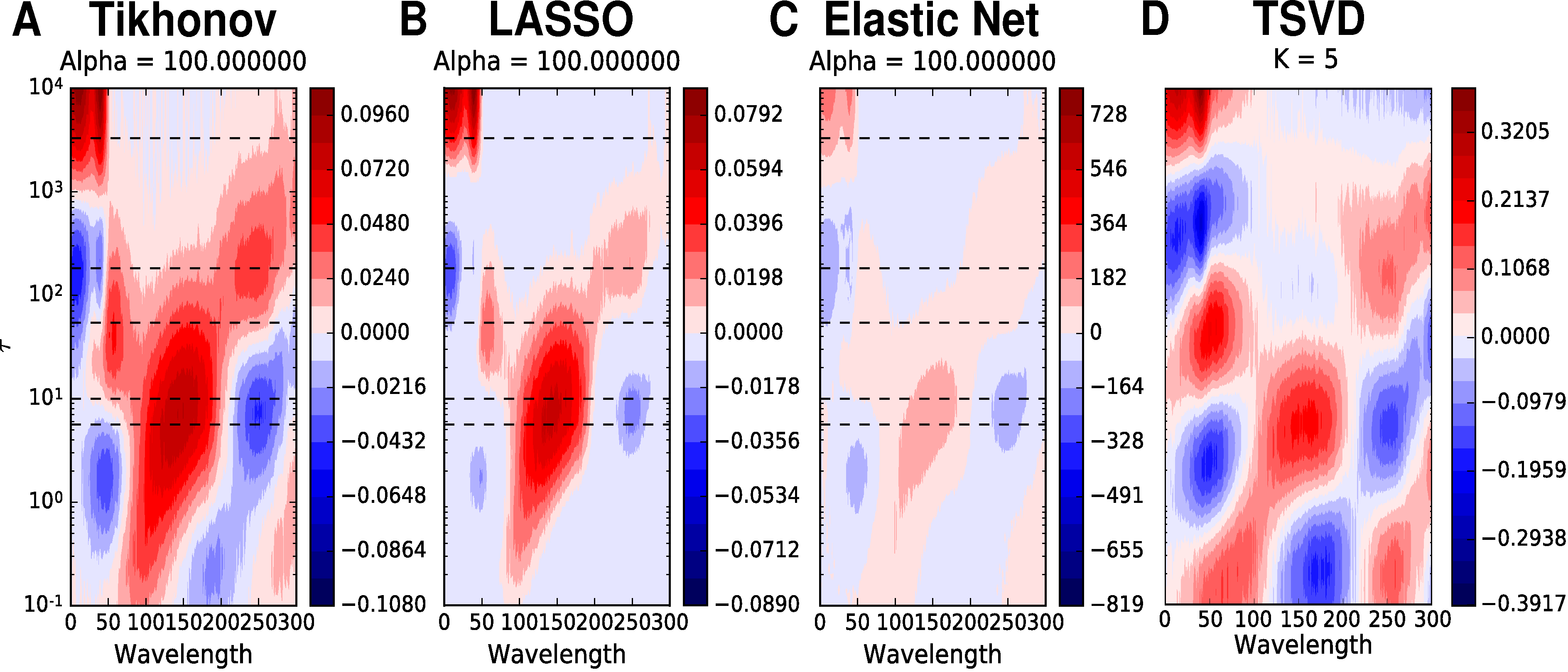

Supplement: S3 Fig — A-D LDMs produced with the indicated regularization routines. Alpha values and K for truncated SVD are also noted. (TIF) [file pcbi.1005528.s003.tif]
