# Supplementary material for: PyLDM - An open source package for lifetime density analysis of time-resolved spectroscopic data
Source: PLoS Comput Biol. 2017 May 22;13(5):e1005528. doi: 10.1371/journal.pcbi.1005528 (PMC5460884; doi:10.1371/journal.pcbi.1005528)
Supplement: S1 Appendix — (PDF) [file pcbi.1005528.s004.pdf]

S1 Appendix

The ordinary least squares solution, as mentioned for Tikhonov regularization, is:

$$\hat{x}_\lambda^{OLS} = (D^T D)^{-1} D^T A_\lambda$$

In the case where D is orthogonal, then $D^T = D^{-1}$, and this simply becomes:

$$\hat{x}_\lambda^{OLS} = D^T A_\lambda \tag{1}$$

Note also that the LASSO minimization problem can be written in expanded form as (note that the vector signs have been removed from x for clarity):

$$\min_x x_\lambda^T D^T D x_\lambda - x_\lambda^T D^T A_\lambda - A_\lambda^T D x_\lambda + A_\lambda^T A_\lambda + \alpha ||Lx||_1$$

which simplifies to:

$$\min_x x_\lambda^T x_\lambda - x_\lambda^T D^T A_\lambda - A_\lambda^T D x_\lambda + A_\lambda^T A_\lambda + \alpha ||Lx||_1 \tag{2}$$

Note that,

$$A_\lambda^T = (x_\lambda^{OLS})^T D^T$$

Using this result and $A_\lambda = D\hat{x}_\lambda^{OLS}$ we arrive at

$$\min_x x_\lambda^T x_\lambda - x_\lambda^T x_\lambda^{OLS} - (x_\lambda^{OLS})^T x_\lambda + \alpha ||Lx||_1$$

Which for a single entry j, using $L = I$, can be written as

$$\min_x x_j^2 - 2x_j^{OLS} x_j + \alpha |x_j| = \min_x O(x) \tag{3}$$

with O simply being the objective function.

This can be split into two scenarios:

1. $x_j^{OLS} \geq 0$: This means that $x_j \geq 0$ because if it were negative, $O(x)$ in (3) could be lowered by making it positive. Now differentiating O w.r.t x_j and setting to zero we get:

$$x_j = 2x_j^{OLS} - \alpha \tag{4}$$

However, as $x_j \geq 0$, then $(2x_j^{OLS} - \alpha) \geq 0$, so we arrive at:

$$x_j = (2x_j^{OLS} - \alpha)^+ = sgn(x_j^{OLS})(2|x_j^{OLS}| - \alpha)^+ \quad (5)$$

2. $x_j^{OLS} \leq 0$: Using the same reasoning this implies that $x_j < 0$, and differentiating will result in the same answer.

Note that the result will hold for $L \neq I$, however, the value of alpha will be multiplied by some function $f(L, x_{i \neq j})$
